# CBCT-Based Assessment of Tooth Morphology and Dental Arch Dimensions Across Skeletal Class I, II, and III Malocclusions: A Cross-Sectional Study

**DOI:** 10.3390/diagnostics16213661

**Published:** 2026-11-05

**Authors:** Fahrettin Kalabalık, Muhammet Can Eren, Emre Haylaz, Orhan Cicek, Ismail Gumussoy, Suayip Burak Duman, Murat Icen, Emre Aytuğar

**Affiliations:** 1 Department of Oral and Maxillofacial Radiology, Faculty of Dentistry, Sakarya University, Sakarya 54100, Türkiye; 2 Department of Orthodontics, Faculty of Dentistry, Zonguldak Bülent Ecevit University, Zonguldak 67600, Türkiye; orhancicek@beun.edu.tr; 3 Department of Oral and Maxillofacial Radiology, Faculty of Dentistry, İnönü University, Malatya 44280, Türkiye; 4 Department of Diagnostic Sciences, Texas A&M College of Dentistry, Texas A&M University, Dallas, TX 75226, USA; 5 Department of Oral and Maxillofacial Radiology, Faculty of Dentistry, Nevşehir Hacı Bektaş Veli University, Nevşehir 50300, Türkiye; 6 Department of Oral and Maxillofacial Radiology, Faculty of Dentistry, Izmir Katip Celebi University, Izmir 35640, Türkiye

**Keywords:** cone-beam computed tomography, dental arch, skeletal malocclusion, tooth morphology

## Abstract

**Background/Objectives**: Three-dimensional data that evaluate tooth morphology and dental arch dimensions together across sagittal skeletal patterns are limited. This cone-beam computed tomography (CBCT) study compared tooth morphology and dental arch dimensions among skeletal Class I, II, and III malocclusions and examined their relationships. **Methods**: This retrospective, cross-sectional study included 111 individuals (37 per group; 24 females and 13 males in each group), selected from archived CBCT records in reverse chronological order with sex-frequency matching. Skeletal class was based on the ANB angle. Crown length, root length, and mesiodistal width of the central incisors, canines, second premolars, and first molars, together with arch perimeter and intercanine, inter-second premolar, and intermolar widths, were measured. Groups were compared using patient-clustered Gaussian generalized estimating equations with Holm-adjusted contrasts and Benjamini–Hochberg false discovery rate (FDR) correction across 35 outcomes; 108 tooth–arch associations were adjusted for class, age, and sex. All analyses were exploratory. **Results**: Five of 35 outcomes showed nominal class effects (*p* < 0.05), mainly in the mandible (central incisor and canine crown lengths, arch perimeter, and intercanine width), but none remained significant after FDR correction (q ≥ 0.169). In exploratory contrasts, mandibular arch perimeter was greater in Class III than in Class I (2.20 mm; 95% CI 0.56–3.85). In the primary models, no mesiodistal width or root length differed nominally. Nine of 108 tooth–arch associations remained significant after FDR correction (standardized β 0.27–0.36), six involving premolar and molar mesiodistal widths. **Conclusions**: No difference in tooth morphology or arch dimensions among skeletal classes remained significant after multiplicity correction; the nominal mandibular findings are exploratory. The findings are descriptive, cannot establish causality, and do not support class-specific treatment recommendations.

## 1. Introduction

Orthodontic treatment aims to establish functional occlusion and facial esthetics. The transverse dimension of the dental arches adapts during growth and treatment, and malocclusion traits affecting the arches are common worldwide [1,2]. Arch dimensions and tooth dimensions together influence tooth alignment and occlusal relationships [3,4].

Skeletal malocclusions, resulting from anteroposterior discrepancies between the maxilla and mandible, are associated with substantial variations in craniofacial morphology. These skeletal differences influence not only maxillomandibular relationships but also dentoalveolar structures and dental arch morphology [5,6]. In response to underlying skeletal disharmonies, dentoalveolar compensation mechanisms may modify tooth position, arch dimensions, and incisor inclination patterns to maintain functional occlusion [7]. Such adaptations may also be accompanied by variations in dental morphology and arch form, highlighting the complex interaction between skeletal pattern, tooth characteristics, and dental arch dimensions.

Dental arch dimensions have traditionally been evaluated using plaster casts and two-dimensional (2D) radiographic techniques [8]. However, these methods are associated with several limitations, including landmark identification errors, superimposition, magnification, and geometric distortion, which may compromise the accurate representation of craniofacial structures [9,10]. Furthermore, conventional approaches provide limited information on the position of the roots within the alveolar bone, because basal bone is assessed through soft-tissue landmarks on casts [11].

In recent years, cone-beam computed tomography (CBCT) has become an important diagnostic and research tool in orthodontics by enabling high-resolution three-dimensional assessment of craniofacial anatomy with less geometric distortion than conventional two-dimensional radiography, although CBCT measurements remain subject to landmark identification and image-quality errors [12,13]. This imaging modality permits the simultaneous evaluation of dental arch dimensions, crown and root morphology, and their spatial relationships [14,15]. Only CBCT examinations already obtained for clinical indications were analyzed in the present study, which does not imply that CBCT should be requested for arch assessment alone.

Although several studies have investigated dental arch dimensions in individuals with different sagittal skeletal patterns, many have relied on two-dimensional analyses or focused on a limited set of dental and arch parameters [4,16]. Studies utilizing three-dimensional CBCT data are still relatively scarce, particularly those evaluating crown morphology, root morphology, and dental arch dimensions simultaneously in individuals with Class I, Class II, and Class III skeletal relationships [17,18,19]. It therefore remains uncertain whether differences in crown or root dimensions accompany sagittal pattern–related differences in arch dimensions, and whether tooth and arch dimensions covary differently in the maxilla and the mandible. Measuring these variables in the same patients, with analyses that account for bilateral measurements within patients, allows this question to be examined directly. The contribution of this study is descriptive and anatomical; clinical applications such as arch-form selection or treatment planning were not tested.

Therefore, the aim of this study was to evaluate tooth morphology and dental arch dimensions in individuals with Class I, Class II, and Class III skeletal malocclusions using CBCT, and to investigate the relationships between these parameters. Because no primary outcome was prespecified, all analyses are reported as exploratory.

## 2. Materials and Methods

### 2.1. Study Design and Ethical Approval

This single-center, retrospective, cross-sectional observational study was conducted using archived cone-beam computed tomography (CBCT) records from the Department of Oral and Maxillofacial Radiology, Faculty of Dentistry, İnönü University, Malatya, Türkiye. Archived CBCT records acquired between January 2020 and December 2024 were screened retrospectively in reverse chronological order against the predefined eligibility criteria (Section 2.5). Selection continued until 37 eligible patients had been included in each sagittal skeletal class, while the sex distribution was kept comparable across the groups (frequency matching): each group comprised 24 females (64.9%) and 13 males (35.1%), giving 111 patients in total. Eligible patients of a sex whose quota in the relevant group had already been filled were not included, so the selection was quota-based rather than strictly consecutive or random. The examinations had been obtained for various clinical indications (for example, orthodontic evaluation), and no additional CBCT scans were obtained for this study. Because the scans were clinically indicated, the sample reflects a referred population and may not be representative of the general population.

The study was approved by the İnönü University Health Sciences Scientific Research Ethics Committee (approval code 2026/9679; approval date 24 March 2026) and conducted according to the Declaration of Helsinki. Written consent was obtained before the CBCT examinations and included authorization for research use of the images; for patients younger than 18 years, consent was obtained from a parent or legal guardian. Patient records were anonymized before analysis. The original CBCT indications for each patient and the numbers of records screened and excluded (with reasons) were not recorded in the analysis dataset; this is acknowledged as a limitation.

### 2.2. Sample Size Calculation and Study Population

The a priori sample size calculation was performed using G*Power software (version 3.1.9.4; Heinrich-Heine-Universität Düsseldorf, Germany), based on the group means and standard deviations reported by Coşkun and Kaya [20] for mandibular lateral incisor root width, which showed the largest standardized difference among the 28 root length and root width comparisons in that study, none of which was statistically significant (means of 6.1, 6.4 and 6.3 mm in skeletal Class I, II and III; common within-group SD 0.4 mm). The resulting Cohen’s f was 0.3118, a medium effect. This variable was not measured in the present study, and the study was not designed to detect smaller differences. For a one-way analysis of variance with three independent groups (an approximation of the final GEE analysis), α = 0.05 and 80% power gave a noncentrality parameter (λ) of 10.2083, a critical F value of 3.0855 and a required total of 105 patients (35 per group). The final sample comprised 111 patients, 37 in each skeletal class, which corresponds to a calculated power of approximately 83% under the same assumptions. The calculation was based on a group-comparison endpoint (root morphology) and did not establish power for the other outcomes or for the exploratory tooth–arch associations. No single primary outcome was formally prespecified; all analyses are therefore interpreted as exploratory, with multiplicity addressed as described in Section 2.9.2.

Maxillary and mandibular central incisors, canines, second premolars and first molars were evaluated bilaterally. The dataset included 222 bilateral observation records per jaw, or 74 observations for each selected tooth type per skeletal class. Right and left tooth measurements were retained separately; generalized estimating equations (GEE) were used to account for repeated observations clustered within patients.

### 2.3. Group Allocation and Demographic Characteristics

Sagittal skeletal classification was based on the ANB angle measured on two-dimensional lateral cephalograms derived from the CBCT volumes [21]. The ANB thresholds were Class I, 0° ≤ ANB ≤ 4°; Class II, ANB > 4°; and Class III, ANB < 0°. Wits appraisal was additionally measured three-dimensionally on the CBCT volumes in 3D Slicer. The occlusal plane was constructed from the interincisal midpoint of the maxillary and mandibular incisors and the central fossae of the right and left maxillary first molars; points A and B were projected perpendicularly onto this plane, and the sagittal distance between the projections (AO–BO) was recorded in millimeters. Wits appraisal was used as a supportive assessment: no numerical Wits thresholds were applied, and when ANB and Wits findings were discordant, the ANB-based classification was used.

Each skeletal group included 24 females (64.9%) and 13 males (35.1%). The mean ages were 19.59 ± 4.23, 19.43 ± 4.41 and 19.52 ± 3.93 years for Class I, II and III, respectively (range, 15.2–37.0 years); no age limits were predefined beyond permanent dentition with completely formed roots. The corresponding mean ANB values were 2.09 ± 0.91°, 5.49 ± 0.99° and −2.95 ± 1.30°, and the mean Wits values were 1.92 ± 2.38, 6.85 ± 1.95 and −6.05 ± 2.04 mm (Table 1).

### 2.4. Selected Teeth and Morphological Standardization

To represent anterior, transitional and posterior dental regions, the maxillary and mandibular central incisors, canines, second premolars and first molars were selected. Morphological standardization was restricted to single-rooted incisors, canines and second premolars; three-rooted maxillary first molars; and two-rooted mandibular first molars. Maxillary first molar mesiobuccal, distobuccal and palatal roots and mandibular first molar mesial and distal roots were assessed separately. Because bilateral presence of all selected teeth with these root configurations was required, patients with an atypical number of roots in any selected tooth were excluded at the patient level; the number of patients excluded for this reason was not recorded. Restriction to these root configurations limits the generalizability of the morphological findings to teeth with typical root anatomy. Comparable CBCT-based methodologies have previously been used to assess crown and root morphology in orthodontic populations and in relation to sagittal skeletal patterns [20,22].

### 2.5. Eligibility Criteria

Patients were included if they met all of the following criteria:

- Permanent dentition;
- A skeletal Class I, II or III sagittal relationship classified using the ANB angle [23] and additionally evaluated using Wits appraisal;
- Bilateral presence of the selected maxillary and mandibular central incisors, canines, second premolars and first molars;
- Complete eruption and completed root formation (closed apices) of the selected teeth;
- Visualization of both dental arches in the CBCT volume;
- Adequate CBCT quality for three-dimensional measurements;
- No history of orthodontic treatment.

Patients were excluded if any of the following were present:

- Missing selected teeth;
- Tooth wear, crown fracture, root resorption, developmental anomalies or conditions that could affect measurements;
- Restorations, prosthetic procedures or endodontic treatment involving selected teeth;
- Impacted, supernumerary or ectopically positioned teeth;
- Craniofacial anomalies, syndromes or jaw pathologies;
- Previous orthognathic surgery or other relevant surgical procedures;
- Inadequate image quality, artefacts or severe CBCT distortion.

Systematic clinical periodontal evaluation was not undertaken in this retrospective image-based study; periodontal conditions that could affect the visibility or identification of the cementoenamel junction (CEJ) could therefore not be assessed independently (see Section 4).

### 2.6. Imaging Protocol and Data Acquisition

All archived examinations were acquired using a NewTom 5G CBCT unit (QR srl, Verona, Italy) under the clinical acquisition protocol (110 kVp; tube current 1–20 mA; field of view 18 × 16 cm; voxel size 0.20–0.25 mm). Data were exported in Digital Imaging and Communications in Medicine (DICOM) format and imported into 3D Slicer (version 5.6.2; MIT, Cambridge, MA, USA). Images were oriented according to natural head position, with separate maxillary and mandibular reconstructions. Axial, coronal and sagittal multiplanar images and three-dimensional renderings were used for measurements; panoramic reconstructions supported anatomical visualization where necessary.

All measurements were made under standardized conditions by one calibrated examiner (M.C.E.). The ANB angle and Wits appraisal were measured first to establish the skeletal classes; the tooth and arch measurements were performed subsequently, on scans whose patient identity and skeletal class labels had been concealed.

### 2.7. Radiographic Measurements

#### 2.7.1. Tooth Morphology Measurements

Crown and root length measurements were performed on sagittal CBCT reconstructions after reorienting each tooth along its long axis. The buccal and palatal cementoenamel junction (CEJ) points in maxillary teeth and the buccal and lingual CEJ points in mandibular teeth were identified. A two-dimensional CEJ reference line was established by connecting these points.tal CBCT reconstructions were performed after reorienting each tooth along its long axis. The buccal and palatal CEJ points in maxillary teeth and the buccal and lingual CEJ points in mandibular teeth were identified. A two-dimensional CEJ reference line was established by connecting these points, and crown and root lengths were measured perpendicular to this reference line.

**Figure 1:**
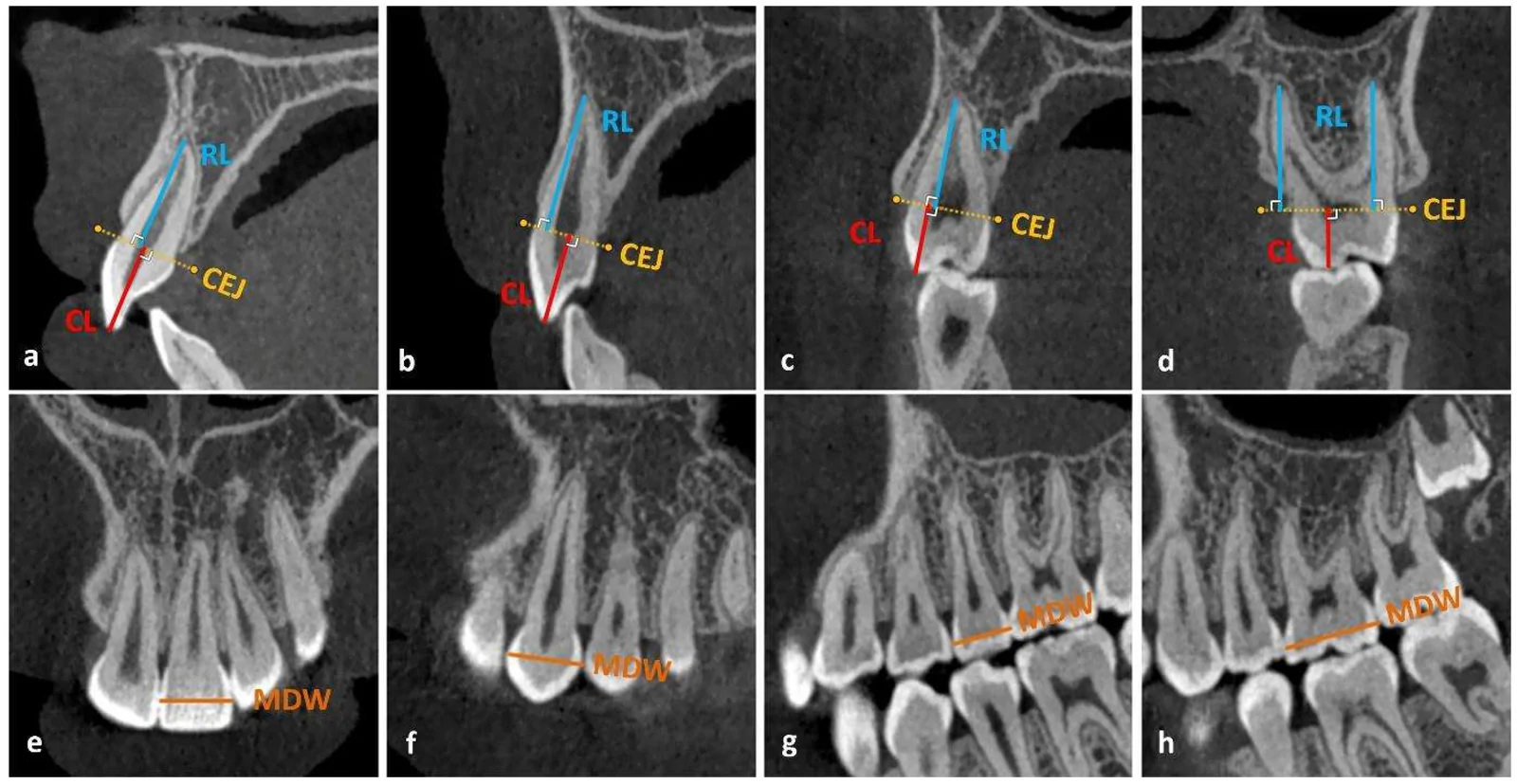
Representative CBCT images illustrating maxillary tooth morphology measurements performed in the study. Images (**a**–**d**) show crown length (CL, red lines) and root length (RL, blue lines) measurements performed on sagittal CBCT reconstructions after reorienting each tooth along its long axis. The yellow dashed line represents the cementoenamel junction (CEJ) reference line established by connecting the buccal and palatal CEJ points. Crown and root lengths were measured perpendicular to this reference line. Mesiodistal width (MDW, orange lines) was measured between the mesial and distal contact points on coronal CBCT sections for anterior teeth (**e**,**f**) and sagittal CBCT sections for posterior teeth (**g**,**h**). Images (**a**,**e**) represent the maxillary central incisor, (**b**,**f**) the maxillary canine, (**c**,**g**) the maxillary second premolar, and (**d**,**h**) the maxillary first molar.

**Figure 2:**
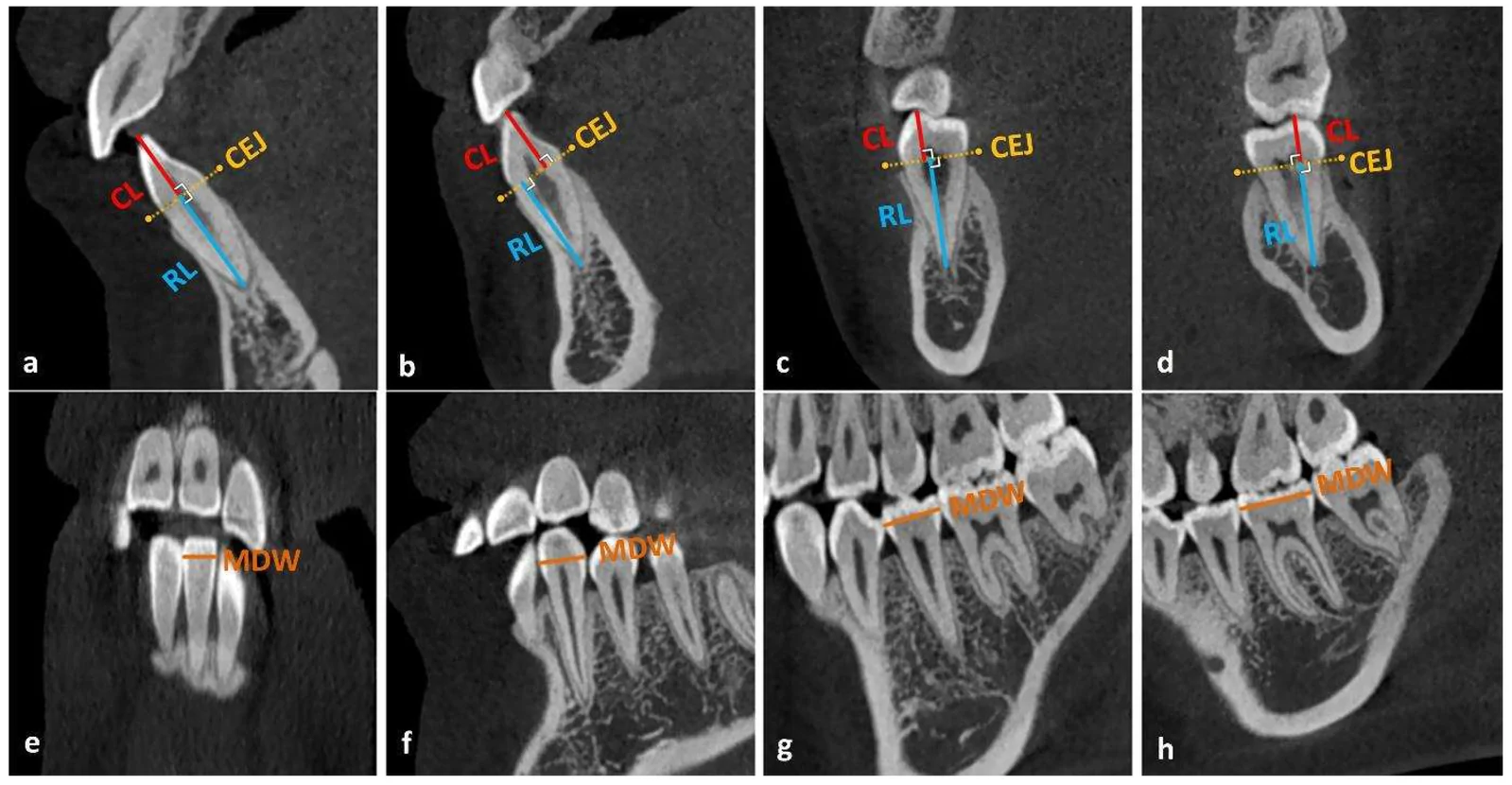
Representative CBCT images illustrating mandibular tooth morphology measurements performed in the study. Images (**a**–**d**) show crown length (CL, red lines) and root length (RL, blue lines) measurements performed on sagittal CBCT reconstructions after reorienting each tooth along its long axis. The yellow dashed line represents the cementoenamel junction (CEJ) reference line established by connecting the buccal and lingual CEJ points. Crown and root lengths were measured perpendicular to this reference line. Mesiodistal width (MDW, orange lines) was measured between the mesial and distal contact points on coronal CBCT sections for anterior teeth (**e**,**f**) and sagittal CBCT sections for posterior teeth (**g**,**h**). Images (**a**,**e**) represent the mandibular central incisor, (**b**,**f**) the mandibular canine, (**c**,**g**) the mandibular second premolar, and (**d**,**h**) the mandibular first molar.

Crown length (CL) was defined as the perpendicular distance from the coronal reference point to the CEJ reference line. The coronal reference point was the incisal edge for incisors, the cusp tip for canines, and the highest cusp tip for premolars and molars [24]. Root length (RL) was defined as the perpendicular distance from the radiographic root apex to the CEJ reference line [25]. Thus, crown and root lengths were measured perpendicular to the CEJ reference line rather than along the longitudinal axes of the tooth or root. In multirooted first molars, each root was evaluated separately using the same measurement principle. The mesiobuccal, distobuccal, and palatal roots of maxillary first molars and the mesial and distal roots of mandibular first molars were measured individually as the perpendicular distance from their respective root apices to the CEJ reference line [26]. Mesiodistal width (MDW) was defined as the distance between the mesial and distal contact points of the tooth. Measurements were performed on coronal CBCT sections for anterior teeth and sagittal CBCT sections for posterior teeth. All measurements were recorded in millimeters. Figure 1 and Figure 2 illustrate the tooth measurements.

#### 2.7.2. Dental Arch Measurements

**Figure 3:**
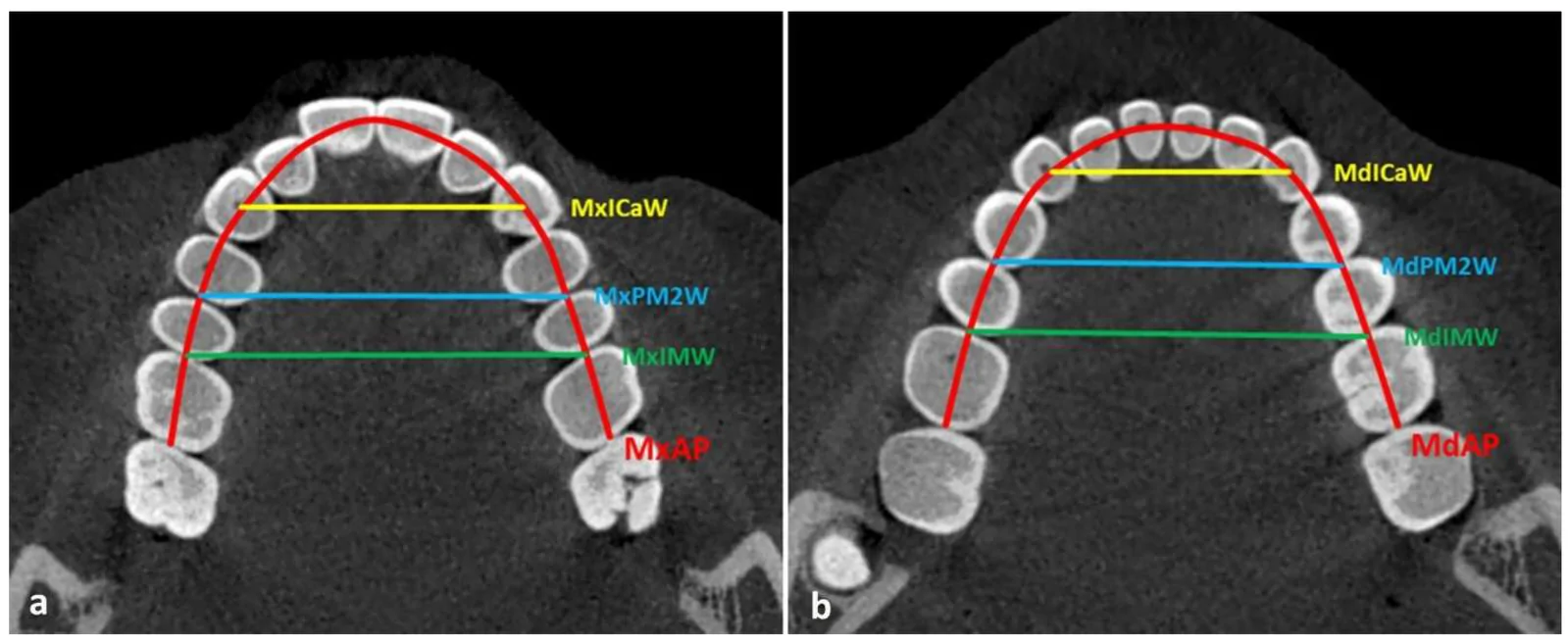
CBCT axial sections illustrating the reference landmarks used for transverse dental arch measurements in the maxillary (**a**) and mandibular (**b**) arches. MxICaW, maxillary intercanine width; MdICaW, mandibular intercanine width; MxPM2W, maxillary inter-second premolar width; MdPM2W, mandibular inter-second premolar width; MxIMW, maxillary intermolar width; MdIMW, mandibular intermolar width; MxAP, maxillary arch perimeter; MdAP, mandibular arch perimeter.

Maxillary and mandibular arch perimeter (MxAP and MdAP) was measured on a single axial CBCT section as the length of a curve drawn with the Curve markup tool of 3D Slicer. The axial section was selected so that the interproximal contact points of the teeth were visualized as clearly as possible. The curve began at the distal border of the first permanent molar on one side, was drawn continuously through the contact points of the teeth visible on the selected section along the dental arch, and ended at the distal border of the first permanent molar on the opposite side (Figure 3). Contact points lying at different vertical levels were not transferred or projected onto a common plane. Arch perimeter is therefore a curvilinear, two-dimensional length measured within one axial plane and not along a three-dimensional curve; in this study, “arch perimeter” refers exclusively to this measurement and not to linear arch depth.

Transverse dimensions were intercanine width (MxICaW/MdICaW), measured between the lingual ridge points of the canines, which were taken as the canine reference landmark; inter-second premolar width (MxPM2W/MdPM2W), measured between the mesial contact points of the second premolars; and intermolar width (MxIMW/MdIMW), measured between the mesial contact points of the first molars. All transverse linear measurements were obtained on axial reconstructions. Each dental arch dimension was recorded once per arch per patient (Figure 3).

### 2.8. Researcher Calibration and Reliability Assessment

Before the study measurements, the examiner underwent landmark and measurement calibration using CBCT datasets outside the analytical sample, under the supervision of an experienced oral and maxillofacial radiologist (E.H.). To assess intra-examiner reliability, 25 randomly selected CBCT scans, representing 22.5% of the total sample, were remeasured by the same examiner after a two-week interval. Inter-examiner reliability was not assessed.

Intra-examiner reliability was evaluated using the intraclass correlation coefficient (ICC) based on a two-way random-effects model (absolute agreement, single measures; ICC (2,1)), and method error was assessed using Dahlberg’s formula [27], E = √[Σd^2^/(2n)], where d is the difference between repeated measurements and n is the number of paired observations. ICC values with 95% confidence intervals and Dahlberg method errors were calculated separately for each of the 35 tooth and arch variables and are summarized by variable group in Table 2.

### 2.9. Statistical Analysis

Descriptive analyses were performed using IBM SPSS Statistics (version 26.0; IBM Corp., Armonk, NY, USA). Generalized estimating equation (GEE) analyses were conducted in Python (version 3.13.5) with statsmodels (version 0.14.6). Continuous data are presented as mean ± standard deviation (SD) and categorical data as frequencies and percentages. Shapiro–Wilk and Levene tests were used to assess distributional characteristics and variance homogeneity; because inference relied on GEE with robust (sandwich) standard errors, which do not require normally distributed outcomes or homogeneous variances, these tests did not determine the choice of analysis. Their results are given in Supplementary Table S1.

#### 2.9.1. Between-Group GEE Models

Bilateral tooth measurements were modeled using Gaussian GEE with an identity link and an independence working correlation structure, with patient identification as the clustering variable and robust sandwich standard errors to account for within-patient dependence. Skeletal class was included as a three-level categorical factor. Overall class effects were tested using robust Wald χ^2^ tests with 2 degrees of freedom. Right and left tooth measurements were not averaged; side was not separately included as a covariate in the primary model. Arch dimensions were analyzed at the patient level (one observation per jaw), using analogous Gaussian marginal models.

#### 2.9.2. Pairwise Comparisons and Multiplicity

For each outcome, the three class contrasts were estimated using model-based Wald tests. Differences and nominal 95% confidence intervals (CIs) were reported, and the three pairwise *p*-values within each outcome were adjusted using the Holm method. Thirty-five omnibus outcomes were evaluated (14 maxillary tooth, 13 mandibular tooth and eight dental arch variables). Benjamini–Hochberg FDR correction was applied jointly to the 35 omnibus *p*-values. Nominal *p* < 0.05 was distinguished from FDR-corrected q < 0.05; findings failing FDR correction were interpreted as exploratory. The confidence intervals are nominal (not adjusted for multiplicity), whereas the pairwise *p*-values are Holm-adjusted; pairwise contrasts are reported only for outcomes with a nominal omnibus *p* < 0.05.

#### 2.9.3. Tooth–Arch Associations

A total of 108 separate GEE association models were fitted (56 maxillary and 52 mandibular). The bilateral tooth variable was the outcome, and the corresponding patient-level arch dimension was the predictor. Both variables were standardized to report standardized regression coefficients (β); skeletal class, age and sex were included as adjustment covariates. Patient-clustered robust standard errors, nominal 95% CIs and two-sided *p*-values were obtained. Benjamini–Hochberg correction was applied across all 108 associations. The coefficients are adjusted standardized regression coefficients and are not Pearson correlation coefficients. Associations were estimated in the pooled sample; differences in association strength between arches or among skeletal classes were not tested formally.

#### 2.9.4. Sensitivity Analyses

The 35 class comparisons were repeated in GEE models adjusted for age and sex; FDR correction was applied across the 35 adjusted omnibus tests, and the results of all adjusted models are given in Supplementary Table S4. The observational, cross-sectional design does not permit causal interpretation.

## 3. Results

### 3.1. Study Population and Intra-Examiner Reliability

The study included 111 individuals (37 per skeletal group), each group comprising 24 women (64.9%) and 13 men (35.1%). Mean ages were 19.59 ± 4.23, 19.43 ± 4.41, and 19.52 ± 3.93 years in skeletal Class I, II, and III groups, respectively (observed range, 15.2–37.0 years). There were 222 bilateral tooth observation records per jaw and 111 individual arch measurements per jaw. Further demographic characteristics are summarized in Table 1.

Intra-examiner reliability, assessed using two repeated CBCT measurements from 25 scans, was good to excellent for all 35 tooth and arch variables (Table 2; values for each variable in Supplementary Table S3). ICC (2,1) values ranged from 0.813 to 0.984 (median 0.925) and were 0.90 or higher for 24 variables. The lowest values were found for maxillary second premolar mesiodistal width (0.813) and maxillary central incisor crown length (0.824); the other nine variables below 0.90 ranged from 0.870 to 0.897. Dahlberg method errors ranged from 0.137 to 0.411 mm, with the largest errors for the distal root of the mandibular first molar (0.411 mm) and the palatal root of the maxillary first molar (0.394 mm).

### 3.2. Tooth Morphology Across Skeletal Classes

#### 3.2.1. Maxillary Tooth Morphology

Maxillary central incisor crown length was the only maxillary outcome with a nominal class effect (Wald χ^2^ = 6.646, df = 2, *p* = 0.036); it was shortest in Class III (9.86 ± 0.91 mm) compared with Class I (10.33 ± 0.80 mm) and Class II (10.30 ± 0.89 mm). This effect did not survive FDR correction (q = 0.252), and none of the pairwise contrasts remained significant after Holm adjustment. Root lengths of the maxillary central incisors (*p* = 0.184) and canines (*p* = 0.074) did not differ nominally among the classes, and none of the 14 maxillary tooth outcomes remained significant after FDR correction (Table 3).

#### 3.2.2. Mandibular Tooth Morphology

Two mandibular outcomes showed nominal class effects: central incisor crown length (Wald χ^2^ = 9.159, df = 2, *p* = 0.010) and canine crown length (Wald χ^2^ = 9.535, df = 2, *p* = 0.009). Central incisor crown length was greater in Class III (8.30 ± 0.69 mm) than in Class I (7.85 ± 0.67 mm; Holm-adjusted difference 0.449 mm, 95% CI 0.143–0.756, *p* = 0.012) and Class II (7.95 ± 0.63 mm; 0.355 mm, 95% CI 0.057–0.653, *p* = 0.039).

Canine crown length was also greater in Class III (8.66 ± 1.09 mm) than in Class I (8.08 ± 0.84 mm; 0.585 mm, 95% CI 0.148–1.021, *p* = 0.017) and Class II (8.03 ± 0.73 mm; 0.628 mm, 95% CI 0.211–1.045, *p* = 0.009). Neither effect survived FDR correction (both q = 0.169). The remaining mandibular outcomes, including second premolar root length (*p* = 0.065) and crown length (*p* = 0.109), showed no nominal class effect (Table 4).

**Table 4:** Descriptive statistics and GEE analysis of mandibular tooth morphology measurements across skeletal classes.

Variable	Class I Mean ± SD	Class II Mean ± SD	Class III Mean ± SD	Wald χ^2^ (df = 2)	*p*	FDR q
MdCIRL	12.92 ± 1.30	12.91 ± 1.25	12.82 ± 1.21	0.144	0.931	0.931
MdCICL	7.85 ± 0.67	7.95 ± 0.63	8.30 ± 0.69	9.159	**0.010**	0.169
MdCIMDW	5.24 ± 0.47	5.31 ± 0.52	5.37 ± 0.54	1.370	0.504	0.715
MdCaRL	16.66 ± 1.96	16.92 ± 1.86	16.20 ± 2.00	2.617	0.270	0.538
MdCaCL	8.08 ± 0.84	8.03 ± 0.73	8.66 ± 1.09	9.535	**0.009**	0.169
MdCaMDW	6.39 ± 0.78	6.33 ± 0.73	6.33 ± 0.68	0.186	0.911	0.931
MdPM2RL	15.15 ± 1.85	15.54 ± 1.61	14.64 ± 1.75	5.459	0.065	0.368
MdPM2CL	6.18 ± 0.61	5.99 ± 0.50	6.24 ± 0.63	4.429	0.109	0.478
MdPM2MDW	6.92 ± 0.50	6.86 ± 0.65	6.82 ± 0.51	0.678	0.713	0.831
MdMMRL	13.10 ± 1.08	13.17 ± 1.19	13.01 ± 1.03	0.403	0.818	0.894
MdMDRL	12.93 ± 1.11	13.04 ± 1.25	12.70 ± 1.14	1.676	0.432	0.688
MdMCL	6.81 ± 0.68	6.66 ± 0.57	6.78 ± 0.48	1.336	0.513	0.715
MdMMDW	10.60 ± 0.58	10.82 ± 0.71	10.66 ± 0.79	2.155	0.341	0.568

Note: Values are mean ± SD (mm); 37 patients and 74 bilateral observations/group for each tooth. Omnibus Wald χ^2^ tests use robust GEE covariance. FDR q-values adjusted across all 35 group outcomes. Md, mandibular; CI, central incisor; Ca, canine; PM2, second premolar; M, first molar; RL, root length; CL, crown length; MDW, mesiodistal width; MMRL, first molar mesial root; MDRL, distal root. Bold type indicates nominal *p* < 0.05; no value reached FDR q < 0.05.

### 3.3. Dental Arch Dimensions

None of the maxillary arch dimensions showed a nominal class effect (arch perimeter *p* = 0.233, intercanine width *p* = 0.191, inter-second premolar width *p* = 0.868, intermolar width *p* = 0.698). Mandibular arch perimeter showed a nominal class effect (Wald χ^2^ = 6.905, df = 2, *p* = 0.032), with means of 85.38 ± 3.36, 86.33 ± 3.61 and 87.59 ± 3.94 mm in Classes I, II and III; Class III exceeded Class I by 2.204 mm (95% CI 0.560–3.848; Holm *p* = 0.026).

Mandibular intercanine width also showed a nominal class effect (Wald χ^2^ = 8.465, df = 2, *p* = 0.015), with means of 24.68 ± 1.90, 24.59 ± 1.47 and 25.55 ± 1.57 mm in Classes I, II and III; Class III exceeded Class II by 0.951 mm (95% CI 0.268–1.635; Holm *p* = 0.019). Neither mandibular arch perimeter (q = 0.252) nor intercanine width (q = 0.169) survived FDR correction. Overall, five of the 35 group comparisons were nominally significant and none survived FDR adjustment (Table 5).

**Table 5:** Descriptive statistics and GEE analysis of maxillary and mandibular dental arch dimensions across skeletal classes.

Variable	Class I Mean ± SD	Class II Mean ± SD	Class III Mean ± SD	Wald χ^2^ (df = 2)	*p*	FDR q
**Maxillary arch**						
MxAP	88.62 ± 3.72	90.28 ± 4.69	89.22 ± 4.13	2.914	0.233	0.538
MxICaW	30.86 ± 2.38	30.29 ± 2.14	29.79 ± 2.77	3.316	0.191	0.538
MxPM2W	36.88 ± 3.02	37.19 ± 3.28	37.23 ± 3.42	0.283	0.868	0.921
MxIMW	41.81 ± 3.17	41.67 ± 3.04	42.28 ± 3.36	0.719	0.698	0.831
**Mandibular arch**						
MdAP	85.38 ± 3.36	86.33 ± 3.61	87.59 ± 3.94	6.905	**0.032**	0.252
MdICaW	24.68 ± 1.90	24.59 ± 1.47	25.55 ± 1.57	8.465	**0.015**	0.169
MdPM2W	35.11 ± 2.26	35.05 ± 2.09	36.04 ± 2.63	3.831	0.147	0.538
MdIMW	40.50 ± 2.83	40.60 ± 2.57	41.59 ± 3.04	3.213	0.201	0.538

Note: Values are mean ± SD (mm), one measurement/patient (n = 37/group). AP, arch perimeter; ICaW, intercanine width; PM2W, inter-second premolar width; IMW, intermolar width. FDR q-values were calculated jointly across the 35 outcomes in Table 3, Table 4 and Table 5. Nominal findings (*p* < 0.05) are exploratory when q ≥ 0.05. Bold type indicates nominal *p* < 0.05; no value reached FDR q < 0.05.

### 3.4. Associations Between Dental Arch Dimensions and Tooth Morphology

A total of 108 tooth–arch associations were examined using patient-clustered Gaussian GEE models adjusted for skeletal class, age and sex; nine remained significant after FDR correction (six maxillary and three mandibular). The coefficients are standardized regression coefficients (β), not Pearson correlation coefficients, and the confidence intervals are nominal and not adjusted for multiplicity. Intervals for all 108 associations are provided in Supplementary Table S2.

#### 3.4.1. Maxillary Arch Associations

The strongest maxillary association was between arch perimeter and first molar mesiodistal width (β = 0.357, 95% CI 0.198–0.516; q = 0.001). First molar mesiodistal width was also associated with inter-second premolar width (β = 0.309, 95% CI 0.143–0.476; q = 0.010) and intermolar width (β = 0.285, 95% CI 0.116–0.453; q = 0.025). Three further associations remained significant: arch perimeter with second premolar mesiodistal width (β = 0.273; q = 0.030), intermolar width with second premolar mesiodistal width (β = 0.278; q = 0.042), and inter-second premolar width with canine crown length (β = 0.269; q = 0.035) (Table 6).

#### 3.4.2. Mandibular Arch Associations

In the mandible, arch perimeter was positively associated with first molar mesiodistal width (β = 0.335, 95% CI 0.172–0.498; q = 0.003), central incisor root length (β = 0.296, 95% CI 0.115–0.476; q = 0.028) and canine crown length (β = 0.290, 95% CI 0.098–0.481; q = 0.041). No association involving mandibular intercanine, inter-second premolar or intermolar width survived correction (Table 7). Among the nine significant associations, six involved mesiodistal widths of premolars or molars (five in the maxillary and one in the mandibular arch), two involved canine crown length (one in each arch), and one involved mandibular central incisor root length. The pattern of significance was not compared formally between the arches.

#### 3.4.3. Age- and Sex-Adjusted Sensitivity Analyses

In the age- and sex-adjusted class models (full results for all 35 outcomes in Supplementary Table S4), nominal effects were found for maxillary central incisor crown length (*p* = 0.026), mandibular central incisor crown length (*p* = 0.005), mandibular canine crown length (*p* = 0.002), mandibular second premolar root length (*p* = 0.035), mandibular arch perimeter (*p* = 0.031) and mandibular intercanine width (*p* = 0.011). None of the 35 adjusted analyses remained significant after FDR correction (all q ≥ 0.079; the smallest was for mandibular canine crown length). Mandibular second premolar root length was the only outcome that became nominally significant only after adjustment (*p* = 0.065 in the primary model), with the shortest roots in Class III (means 14.64 versus 15.15 and 15.54 mm in Classes I and II; Table 4). The observed group differences are therefore exploratory rather than definitive.

## 4. Discussion

In the present study, tooth morphology and dental arch dimensions were evaluated three-dimensionally using CBCT images in individuals with different sagittal skeletal patterns. Of 35 group comparisons, five were nominally significant (maxillary central incisor crown length; mandibular central incisor crown length, canine crown length, arch perimeter and intercanine width), but none remained significant after FDR correction, and none of the mesiodistal width or root length comparisons were nominally significant. The nominal differences should therefore be regarded as hypothesis-generating. In contrast, nine of 108 adjusted tooth–arch associations remained significant after FDR correction.

Compatibility between the maxillary and mandibular tooth dimensions is important for achieving an ideal occlusal relationship [28,29], and tooth dimensions have been widely studied [30]. Three-dimensional models derived from CBCT data have been reported to provide tooth-size measurements with accuracy and reproducibility comparable to digital models obtained from study casts [10,31].

No significant differences were observed among skeletal classes in the mesiodistal widths of maxillary or mandibular teeth. This absence of detected differences should not be read as proof of equivalence, because the study was not designed or powered to demonstrate equivalence; it is, however, compatible with tooth widths being relatively stable characteristics shaped largely by genetic and developmental factors during odontogenesis [32,33]. The results are consistent with those of Araujo and Souki [34], who found no significant differences in the mesiodistal widths of individual anterior teeth among Angle classes, although the anterior Bolton ratio, a proportional measure, was higher in Class III. Limited differences in some tooth groups, such as the mandibular first molar between Class I and Class II malocclusion in a cast-based study, have also been reported [28] and may reflect differences in sample characteristics, ethnic background, methods, and the teeth examined.

Root morphology, together with the magnitude of the applied force, the type of tooth movement, and treatment duration, may contribute to orthodontic root resorption [35,36]. Short, blunt, or pipette-shaped roots have been associated with greater root resorption during treatment, and root length influences the initial displacement pattern of the tooth [37,38]; among the maxillary incisors, central incisors were reported to be more prone to marked root resorption before orthodontic treatment [39]. In the present study, root length differences among skeletal classes were small and inconsistent. Maxillary central incisor, maxillary canine, and mandibular second premolar root lengths (*p* = 0.184, 0.074, and 0.065, respectively) did not differ nominally in the primary models; mandibular second premolar root length reached nominal significance only in the age- and sex-adjusted model (*p* = 0.035; shortest in Class III), and no root length comparison was significant after FDR correction. This is in line with Coşkun and Kaya [20], who found no significant differences in root length or root width among skeletal Class I, II and III groups of 20 patients each. Our data therefore do not support class-specific root length differences or class-specific biomechanical recommendations; because root resorption was not evaluated, the results concern baseline root morphology only.

Dental arch dimensions are important parameters in orthodontic treatment planning for determining arch form [40]. No significant differences were observed among skeletal classes in any of the maxillary arch measurements, and no arch measurement differed significantly between Class I and Class II. Descriptively, Class II individuals showed larger maxillary and mandibular arch perimeters and smaller maxillary and mandibular intercanine widths than Class I individuals, but the differences were small (for example, mandibular intercanine width 24.59 versus 24.68 mm). Maxillary intercanine width was lowest in Class III (29.79 mm) and highest in Class I (30.86 mm), with Class II intermediate (30.29 mm).

Fu et al. [41] reported increased arch length and a narrower anterior arch segment in skeletal Class II individuals, and Zhang et al. [14] compared basal arch widths between normal occlusion and Class II malocclusion using CBCT. These studies are not directly comparable with ours, because they differed in the structures measured (basal versus dental arch), the reference group (normal occlusion versus Class I), sample characteristics, and methods; in our dental measurements, Class II values did not differ significantly from those of Class I. Lee [42] noted that changes introduced in the intercanine region may not be maintained in the long term, but treatment response and stability were not assessed in the present study.

In Class III individuals, maxillary arch perimeter and posterior arch widths were descriptively greater, and maxillary intercanine width lower, than in Class I, but these differences were not statistically significant. In the mandible, all arch parameters were descriptively higher in Class III, and arch perimeter (Class III–I: 2.20 mm, 95% CI 0.56–3.85; Holm *p* = 0.026) and intercanine width (Class III–II: 0.95 mm, 95% CI 0.27–1.64; Holm *p* = 0.019) showed nominal differences that did not survive FDR correction (q = 0.252 and 0.169, respectively). These exploratory findings suggest, at most, a tendency toward a longer and wider anterior mandibular arch in Class III individuals. They are consistent in direction with Suk et al. [11], who reported increased mandibular dental and basal intercanine widths in Class III individuals compared with normal occlusion in a CBCT-based study, with the increase in transverse width particularly evident in the anterior region.

The mandibular pattern of nominally greater arch dimensions and crown lengths in Class III, without differences in mesiodistal tooth widths, should be interpreted with attention to the distinction between dentoalveolar adaptation and intrinsic tooth morphology. Tooth width is established during odontogenesis [32,33], whereas arch perimeter and transverse arch widths depend on tooth position and alveolar bone and may change with compensatory tooth movement in response to the underlying skeletal discrepancy [7]. Differences in arch dimensions without differences in mesiodistal width are therefore more compatible with dentoalveolar adaptation than with differences in intrinsic tooth size. Crown length, measured here from the incisal edge or cusp tip to the cementoenamel junction (CEJ), is an anatomical dimension and, in principle, does not depend on the degree of eruption or on the gingival margin. It can nevertheless be influenced by slight incisal or cuspal wear that escaped detection, by the accuracy with which the CEJ and the incisal edge or cusp tip are identified at a voxel size of 0.20–0.25 mm, and by measurement error; our design cannot separate true morphological differences from these sources. The nominal mandibular differences (about 0.35–0.63 mm) exceeded the Dahlberg errors of the corresponding variables (0.19 mm for the central incisor and 0.24 mm for the canine; Supplementary Table S3), but they did not survive FDR correction (q = 0.169). Moreover, maxillary central incisor crown length was nominally shorter in Class III (9.86 mm) than in Class I and II (10.33 and 10.30 mm), opposite in direction to the mandibular findings, which argues against a uniform effect of skeletal class on crown length.

From a clinical perspective, the present data are descriptive and do not allow recommendations about arch-form modification; movements beyond the limits of the alveolar bone have been associated with dehiscence and fenestration [43], but this was not examined here.

After adjustment for skeletal class, age, and sex, nine tooth–arch associations remained significant after FDR correction, all positive and of small to moderate magnitude (standardized β = 0.27–0.36). Five of the six maxillary associations involved mesiodistal widths of premolars and molars (the largest being between maxillary arch perimeter and first molar mesiodistal width, β = 0.357), and the sixth involved canine crown length; in the mandible, arch perimeter was associated with first molar mesiodistal width (β = 0.335), canine crown length, and central incisor root length. The significant associations involved both anterior and posterior teeth in both arches, and because different patterns of statistical significance do not establish that the associations themselves differ, no between-arch comparison of association strength can be made. Arch perimeter, measured as a curvilinear length passing through the contact points of the teeth, is geometrically related to the mesiodistal widths of the teeth along its path; because the curve starts at the distal border of the first molars, it includes the full mesiodistal width of both first molars. Its associations with mesiodistal widths are therefore partly anatomical and should not be interpreted as evidence of biological coupling alone; transverse widths measured between mesial contact points are likewise related to the widths of the teeth mesial to those points. Paulino et al. [44] found, in a cast-based study, a very high correlation between arch perimeter (termed arch length in that study) and intercanine width (r = 0.925) but only low correlations between arch perimeter and intermolar width (r = 0.43–0.47), indicating that the strength of association varies among arch parameters. The small-to-moderate coefficients indicate that arch dimensions cannot be explained by tooth morphology alone; craniofacial growth pattern, alveolar bone development, and dentoalveolar adaptation are likely to contribute [32,33].

Vertical skeletal pattern may confound both the transverse arch widths and the dentoalveolar dimensions examined here. Transverse arch dimensions have been reported to differ according to growth pattern [4], and vertical pattern is also related to incisor inclination, alveolar dimensions, and the available arch space. Because vertical pattern, incisor inclination, and crowding were neither recorded nor adjusted for, they could have inflated or masked associations between tooth and arch dimensions and contributed to the small-to-moderate coefficients; the adjusted models accounted only for skeletal class, age, and sex.

The main strength of this study is the CBCT-based assessment of tooth morphology and dental arch dimensions in the same patients, using statistical methods that account for bilateral measurements within patients and multiple comparisons. Although CBCT provides three-dimensional imaging data, arch perimeter measurements were performed two-dimensionally on a single reconstructed CBCT section. Therefore, the present study should be regarded as a CBCT-based morphometric assessment rather than a fully three-dimensional analysis of all measured parameters. Several limitations should be considered. The cross-sectional design does not allow causal inference. The study was single-center and retrospective, and the CBCT examinations had been obtained for clinical indications, so the sample reflects a referred population. The numbers of records screened and excluded were not recorded, and patients were selected in reverse chronological order with sex quotas rather than by random or strictly consecutive sampling; selection bias therefore cannot be excluded, the female-to-male ratio (about 65%:35%) results from the matching procedure, and the periods from which patients were drawn may differ between groups. The age range was wide (15.2–37.0 years), and residual growth may have influenced arch dimensions despite adjustment for age. Inclusion was restricted to patients with all selected teeth present bilaterally and to specific root configurations, which limits the generalizability of the morphological findings to teeth with typical anatomy. Periodontal status, vertical skeletal pattern, incisor inclination, and crowding were not assessed, and Wits appraisal was used only as a supportive measure without numerical cutoff values (ANB took precedence in discordant cases). All measurements were made by one examiner, who had also established the skeletal classes; patient identity and class labels were concealed during the tooth and arch measurements, but full blinding cannot be assured, because the facial skeleton visible in CBCT volumes may have allowed inference of skeletal pattern. Only intra-examiner reliability was assessed on 25 scans; the ICC was good rather than excellent (0.81–0.90) for 11 of the 35 variables, and the confidence intervals of several ICCs were wide (Supplementary Table S3). Arch perimeter was measured on a single axial section through the contact points visible on that section, and contact points at different vertical levels were not transferred to a common plane; it is therefore a two-dimensional measurement that may not fully reflect the three-dimensional morphology of the dental arch, particularly when teeth are tilted or crowded. The contact points were not saved as separate landmarks, which may limit exact replication of this measurement. Arch widths describe dental rather than basal arch width. Mesiodistal widths of lateral incisors and first premolars, which also lie along the arch-perimeter path, were not measured, so the inference of dentoalveolar adaptation applies only to the four measured tooth types. The nominal mandibular crown length differences (about 0.35–0.63 mm) were about two to three times the Dahlberg errors of the corresponding variables (0.19 mm for the central incisor and 0.24 mm for the canine; Supplementary Table S3), but measurement error, including a systematic class-related error in locating the CEJ and cusp tip, cannot be excluded as a contributor, particularly at a voxel size of 0.20–0.25 mm and without full blinding. Finally, the study was not powered for the tooth–arch associations; the group differences were small and none survived FDR correction, and alveolar bone thickness and height were not measured. Future three-dimensional studies in larger samples that include different ethnic groups, vertical skeletal pattern, inter-examiner reliability, and basal bone morphology are needed to confirm or refute these exploratory findings.

## 5. Conclusions

In this exploratory CBCT study, no difference in tooth morphology or dental arch dimensions among skeletal Class I, II, and III groups remained significant after multiplicity correction. Nominal differences were confined mainly to the mandible, with a tendency toward greater central incisor and canine crown lengths, greater arch perimeter, and greater intercanine width in Class III individuals; no arch measurement distinguished Class I from Class II. Mesiodistal tooth widths, particularly those of premolars and molars, showed small-to-moderate positive associations with arch perimeter and transverse arch widths after adjustment for skeletal class, age, and sex, without a clear anterior–posterior division between the arches. These descriptive findings are hypothesis-generating; treatment response and stability were not assessed, and no treatment recommendations can be drawn from them.

## Figures and Tables

**Table 1:** Demographic and skeletal characteristics of the study groups.

Characteristics	Class I (n = 37)	Class II (n = 37)	Class III (n = 37)
**Age (years), mean ± SD**	19.59 ± 4.23	19.43 ± 4.41	19.52 ± 3.93
**Sex, n (%)**			
Female	24 (64.9)	24 (64.9)	24 (64.9)
Male	13 (35.1)	13 (35.1)	13 (35.1)
**Skeletal, mean ± SD**			
ANB (°)	2.09 ± 0.91	5.49 ± 0.99	−2.95 ± 1.30
Wits appraisal (mm)	1.92 ± 2.38	6.85 ± 1.95	−6.05 ± 2.04

Note: SD, standard deviation; n, number of patients; mm, millimeters.

**Table 2:** Intra-examiner reliability and method error of CBCT measurements, summarized by variable group.

Variable Group	Variables (n)	ICC (2,1), Range	Dahlberg Error (mm), Range
Crown length, maxillary teeth	4	0.824–0.979	0.164–0.293
Crown length, mandibular teeth	4	0.870–0.939	0.180–0.237
Root length, maxillary teeth	6	0.897–0.984	0.205–0.394
Root length, mandibular teeth	5	0.912–0.980	0.244–0.411
Mesiodistal width, maxillary teeth	4	0.813–0.973	0.137–0.255
Mesiodistal width, mandibular teeth	4	0.883–0.933	0.163–0.296
Dental arch dimensions, maxilla	4	0.916–0.944	0.170–0.213
Dental arch dimensions, mandible	4	0.888–0.910	0.201–0.268
All variables	35	0.813–0.984	0.137–0.411

Note: ICC, intraclass correlation coefficient. ICC values were calculated using a two-way random-effects model for absolute agreement based on single measurements [ICC (2,1)]. Intra-examiner reliability was assessed using two repeated measurements of 25 scans. Entries show the lowest and highest values among the variables in each group. Tooth variables comprise the central incisor, canine, second premolar and first molar (each root of the first molar measured separately for root length); arch dimensions comprise arch perimeter and intercanine, inter-second premolar and intermolar widths. Dahlberg method errors are expressed in millimeters. The ICC, 95% confidence interval and Dahlberg error of each of the 35 variables are given in Supplementary Table S3.

**Table 3:** Descriptive statistics and GEE analysis of maxillary tooth morphology measurements across skeletal classes.

Variable	Class I Mean ± SD	Class II Mean ± SD	Class III Mean ± SD	Wald χ^2^ (df = 2)	*p*	FDR q
MxCIRL	12.98 ± 1.61	13.58 ± 1.43	13.11 ± 1.51	3.388	0.184	0.538
MxCICL	10.33 ± 0.80	10.30 ± 0.89	9.86 ± 0.91	6.646	**0.036**	0.252
MxCIMDW	8.42 ± 0.71	8.53 ± 0.73	8.25 ± 0.83	2.379	0.304	0.538
MxCaRL	17.54 ± 2.33	17.79 ± 2.38	16.60 ± 2.39	5.221	0.074	0.368
MxCaCL	8.45 ± 0.95	8.34 ± 0.85	8.55 ± 1.22	0.838	0.658	0.822
MxCaMDW	7.13 ± 0.74	7.31 ± 0.80	7.04 ± 0.64	2.647	0.266	0.538
MxPM2RL	13.53 ± 1.72	13.94 ± 1.75	14.06 ± 1.25	2.359	0.307	0.538
MxPM2CL	5.83 ± 0.66	5.67 ± 0.59	5.90 ± 0.58	3.069	0.216	0.538
MxPM2MDW	6.48 ± 0.45	6.53 ± 0.50	6.36 ± 0.47	2.520	0.284	0.538
MxMMRL	11.73 ± 1.22	12.04 ± 1.41	12.15 ± 1.19	2.464	0.292	0.538
MxMDRL	11.77 ± 1.09	11.80 ± 1.45	11.95 ± 1.18	0.517	0.772	0.872
MxMPRL	12.75 ± 1.03	12.97 ± 1.27	12.96 ± 1.25	0.929	0.629	0.815
MxMCL	6.96 ± 0.98	7.04 ± 0.63	7.18 ± 0.69	1.497	0.473	0.715
MxMMDW	9.93 ± 0.61	10.06 ± 0.72	9.87 ± 0.87	1.267	0.531	0.715

Note: Values are mean ± SD (mm). n = 37 patients/group and 74 bilateral tooth observations/group for each selected tooth type. Gaussian GEE with patient-clustered robust standard errors; omnibus class effect: Wald χ^2^ (df = 2). Benjamini–Hochberg FDR correction covers 35 outcomes in Table 3, Table 4 and Table 5. Mx, maxillary; CI, central incisor; Ca, canine; PM2, second premolar; M, first molar; RL, root length; CL, crown length; MDW, mesiodistal width; MMRL, first molar mesiobuccal root; MDRL, distobuccal root; MPRL, palatal root. Bold type indicates nominal *p* < 0.05; no value reached FDR q < 0.05.

**Table 6:** Adjusted GEE associations between maxillary arch dimensions and tooth morphology.

Tooth Variable	MxAP β (FDR q)	MxICaW β (FDR q)	MxPM2W β (FDR q)	MxIMW β (FDR q)
MxCIRL	0.187 (0.169)	0.004 (0.964)	0.142 (0.402)	0.192 (0.187)
MxCICL	0.073 (0.596)	0.152 (0.371)	0.140 (0.371)	0.108 (0.498)
MxCIMDW	0.238 (0.059)	0.207 (0.171)	0.135 (0.371)	0.173 (0.169)
MxCaRL	0.179 (0.165)	0.187 (0.262)	0.119 (0.475)	0.120 (0.475)
MxCaCL	0.193 (0.126)	0.112 (0.437)	0.269 (0.035)	0.243 (0.070)
MxCaMDW	0.168 (0.271)	0.252 (0.059)	0.091 (0.590)	0.133 (0.402)
MxPM2RL	0.129 (0.455)	0.174 (0.144)	0.120 (0.475)	0.078 (0.662)
MxPM2CL	0.051 (0.735)	−0.012 (0.937)	−0.023 (0.917)	−0.016 (0.937)
MxPM2MDW	0.273 (0.030)	0.179 (0.165)	0.206 (0.154)	0.278 (0.042)
MxMMRL	0.084 (0.680)	0.011 (0.937)	0.037 (0.870)	0.017 (0.937)
MxMDRL	0.083 (0.727)	−0.031 (0.884)	0.016 (0.937)	−0.012 (0.937)
MxMPRL	0.051 (0.761)	−0.060 (0.735)	−0.046 (0.781)	−0.109 (0.498)
MxMCL	0.127 (0.391)	0.082 (0.684)	0.124 (0.426)	0.222 (0.086)
MxMMDW	0.357 (0.001)	0.279 (0.062)	0.309 (0.010)	0.285 (0.025)

Note: Entries are standardized β (Benjamini–Hochberg FDR q), from patient-clustered GEE models adjusted for skeletal class, age and sex (n = 111 patients; 222 tooth observations). The FDR family comprises 108 maxillary and mandibular associations. A q < 0.05 indicates FDR significance. Nominal 95% CIs for all 108 associations are given in Supplementary Table S2. AP denotes arch perimeter; other abbreviations are defined in Table 3 and Table 5.

**Table 7:** Adjusted GEE associations between mandibular arch dimensions and tooth morphology.

Tooth Variable	MdAP β (FDR q)	MdICaW β (FDR q)	MdPM2W β (FDR q)	MdIMW β (FDR q)
MdCIRL	0.296 (0.028)	0.089 (0.651)	0.238 (0.070)	0.239 (0.070)
MdCICL	0.165 (0.340)	0.139 (0.402)	0.196 (0.144)	0.163 (0.219)
MdCIMDW	0.255 (0.070)	0.122 (0.475)	0.070 (0.680)	−0.007 (0.946)
MdCaRL	0.026 (0.911)	−0.069 (0.755)	0.041 (0.832)	−0.060 (0.735)
MdCaCL	0.290 (0.041)	0.100 (0.475)	0.132 (0.402)	0.117 (0.475)
MdCaMDW	0.134 (0.426)	0.067 (0.763)	−0.055 (0.770)	−0.074 (0.662)
MdPM2RL	0.054 (0.735)	−0.013 (0.937)	0.071 (0.646)	0.019 (0.911)
MdPM2CL	0.025 (0.911)	−0.133 (0.475)	−0.144 (0.371)	−0.114 (0.475)
MdPM2MDW	0.169 (0.169)	0.015 (0.937)	0.067 (0.662)	0.114 (0.426)
MdMMRL	0.105 (0.596)	−0.053 (0.791)	0.074 (0.735)	0.034 (0.894)
MdMDRL	0.170 (0.371)	0.014 (0.937)	0.088 (0.663)	0.053 (0.791)
MdMCL	0.218 (0.069)	0.064 (0.735)	0.101 (0.511)	0.091 (0.596)
MdMMDW	0.335 (0.003)	0.073 (0.662)	0.080 (0.596)	0.078 (0.596)

Note: Entries are standardized β (FDR q) from multivariable Gaussian GEE models adjusted for skeletal class, age and sex. n = 111 patients, 222 bilateral tooth observations; 108 total association tests corrected jointly. For all 108 associations, 95% CIs are given in Supplementary Table S2. AP denotes arch perimeter.

## Data Availability

The data presented in this study are available on reasonable request from the corresponding author. The data are not publicly available because of patient privacy restrictions.

## Supplementary Materials

The following supporting information can be downloaded at: https://www.mdpi.com/article/10.3390/diagnostics16213661/s1, Table S1: Shapiro–Wilk and Levene test results for the 35 outcomes; Table S2: Standardized coefficients with nominal 95% confidence intervals for all 108 tooth–arch associations; Table S3: Intra-examiner reliability (ICC (2,1) with 95% confidence interval and Dahlberg error) for each of the 35 tooth and arch variables; Table S4: Age- and sex-adjusted GEE sensitivity analyses (Wald χ^2^, *p*-value and FDR q-value) of all 35 outcomes across skeletal classes.
