# Supplementary material for: CBCT-Based Assessment of Tooth Morphology and Dental Arch Dimensions Across Skeletal Class I, II, and III Malocclusions: A Cross-Sectional Study
Source: Diagnostics (Basel). 2026 Nov 5;16(21):3661. doi: 10.3390/diagnostics16213661 (PMC13761849; doi:10.3390/diagnostics16213661)
Supplement: Supplementary file 1 [file diagnostics-16-03661-s001.zip › diagnostics-4647311-supplementary.pdf]

Supplementary Tables (to be supplied as online supporting information)

Supplementary Table S1. Shapiro–Wilk and Levene test results for the 35 outcomes

Outcome	Shapiro–Wilk p, Class I	Shapiro–Wilk p, Class II	Shapiro–Wilk p, Class III	Levene p
MxCIRL	**0.025**	0.076	0.059	0.581
MxCICL	0.294	0.453	0.256	0.585
MxCIMDW	0.075	**0.011**	0.835	0.221
MxCaRL	0.249	0.131	0.142	0.878
MxCaCL	**0.010**	**0.017**	**0.020**	0.093
MxCaMDW	**0.018**	0.069	**0.029**	0.052
MxPM2RL	0.219	0.246	**0.007**	**0.002**
MxPM2CL	0.303	0.061	**0.001**	0.354
MxPM2MDW	0.151	0.083	**<0.001**	0.455
MxMMRL	**<0.001**	0.300	**0.018**	0.284
MxMDRL	**0.030**	**0.001**	0.450	0.051
MxMPRL	0.722	0.120	**0.023**	0.226
MxMCL	**<0.001**	**0.006**	0.559	0.051
MxMMDW	**0.015**	0.148	0.413	**0.012**
MxAP	0.281	**0.049**	0.698	0.560
MxICaW	0.465	0.258	0.435	0.149
MxPM2W	0.324	0.054	0.583	0.616
MxIMW	0.822	0.134	0.160	0.686
MdCIRL	0.102	**<0.001**	0.160	0.574
MdCICL	0.093	**0.024**	0.138	0.824
MdCIMDW	0.072	0.198	**<0.001**	0.619
MdCaRL	0.337	0.105	0.163	0.560
MdCaCL	0.424	0.302	**0.002**	**0.003**
MdCaMDW	**0.020**	**<0.001**	0.089	0.535
MdPM2RL	**0.008**	**<0.001**	0.060	0.542
MdPM2CL	0.114	0.090	0.185	0.086
MdPM2MDW	0.829	**0.007**	0.212	0.372
MdMMRL	**0.039**	0.101	0.536	0.533
MdMDRL	0.593	**0.010**	0.100	0.615
MdMCL	0.123	0.098	0.531	**0.002**
MdMMDW	**<0.001**	0.175	0.194	**0.015**
MdAP	0.077	0.972	0.196	0.968
MdICaW	0.584	0.413	0.337	0.316
MdPM2W	0.607	0.268	0.721	0.527
MdIMW	0.826	0.106	0.256	0.555

Note: Values are p-values. Shapiro–Wilk tests were applied within each skeletal class (74 bilateral observations per class for tooth variables; 37 patients per class for arch variables); Levene's test (mean-centred) compared variances across the three classes. Because inference was based on GEE with patient-clustered robust standard errors, these tests were descriptive and did not determine the choice of analysis. Bold type indicates $p < 0.05$. Abbreviations are defined in Tables 3–5.

Supplementary Table S2. Standardized β with nominal 95% confidence intervals for all 108 tooth–arch associations

Arch–tooth association	Std. β	Nominal 95% CI	p	FDR q
MxAP–MxCIRL	0.187	0.007 to 0.367	0.042	0.169
MxAP–MxCICL	0.073	-0.083 to 0.228	0.358	0.596
MxAP–MxCIMDW	0.238	0.069 to 0.407	0.006	0.059
MxAP–MxCaRL	0.179	0.011 to 0.346	0.036	0.165
MxAP–MxCaCL	0.193	0.028 to 0.359	0.022	0.126
MxAP–MxCaMDW	0.168	-0.020 to 0.356	0.080	0.271
MxAP–MxPM2RL	0.129	-0.069 to 0.328	0.202	0.455
MxAP–MxPM2CL	0.051	-0.120 to 0.222	0.558	0.735
MxAP–MxPM2MDW	0.273	0.103 to 0.443	0.002	**0.030**
MxAP–MxMMRL	0.084	-0.142 to 0.311	0.466	0.680
MxAP–MxMDRL	0.083	-0.164 to 0.329	0.512	0.727
MxAP–MxMPRL	0.051	-0.135 to 0.237	0.592	0.761
MxAP–MxMCL	0.127	-0.042 to 0.296	0.141	0.391
MxAP–MxMMDW	0.357	0.198 to 0.516	<0.001	**0.001**
MxICaW–MxCIRL	0.004	-0.180 to 0.188	0.964	0.964
MxICaW–MxCICL	0.152	-0.041 to 0.344	0.123	0.371
MxICaW–MxCIMDW	0.207	0.005 to 0.408	0.044	0.171
MxICaW–MxCaRL	0.187	-0.019 to 0.393	0.075	0.262
MxICaW–MxCaCL	0.112	-0.056 to 0.281	0.190	0.437
MxICaW–MxCaMDW	0.252	0.072 to 0.431	0.006	0.059
MxICaW–MxPM2RL	0.174	0.019 to 0.329	0.027	0.144
MxICaW–MxPM2CL	-0.012	-0.206 to 0.183	0.906	0.937
MxICaW–MxPM2MDW	0.179	0.011 to 0.347	0.037	0.165
MxICaW–MxMMRL	0.011	-0.192 to 0.214	0.915	0.937
MxICaW–MxMDRL	-0.031	-0.226 to 0.163	0.753	0.884
MxICaW–MxMPRL	-0.060	-0.263 to 0.142	0.558	0.735
MxICaW–MxMCL	0.082	-0.142 to 0.306	0.475	0.684
MxICaW–MxMMDW	0.279	0.077 to 0.482	0.007	0.062
MxPM2W–MxCIRL	0.142	-0.055 to 0.339	0.157	0.402
MxPM2W–MxCICL	0.140	-0.041 to 0.321	0.129	0.371
MxPM2W–MxCIMDW	0.135	-0.040 to 0.309	0.131	0.371
MxPM2W–MxCaRL	0.119	-0.082 to 0.319	0.246	0.475
MxPM2W–MxCaCL	0.269	0.096 to 0.442	0.002	**0.035**
MxPM2W–MxCaMDW	0.091	-0.091 to 0.272	0.328	0.590
MxPM2W–MxPM2RL	0.120	-0.074 to 0.314	0.226	0.475
MxPM2W–MxPM2CL	-0.023	-0.223 to 0.177	0.823	0.917
MxPM2W–MxPM2MDW	0.206	0.018 to 0.395	0.031	0.154
MxPM2W–MxMMRL	0.037	-0.178 to 0.253	0.733	0.870
MxPM2W–MxMDRL	0.016	-0.213 to 0.245	0.891	0.937
MxPM2W–MxMPRL	-0.046	-0.231 to 0.140	0.629	0.781
MxPM2W–MxMCL	0.124	-0.057 to 0.306	0.180	0.426
MxPM2W–MxMMDW	0.309	0.143 to 0.476	<0.001	**0.010**
MxIMW–MxCIRL	0.192	-0.000 to 0.384	0.050	0.187
MxIMW–MxCICL	0.108	-0.081 to 0.298	0.263	0.498
MxIMW–MxCIMDW	0.173	0.006 to 0.340	0.042	0.169
MxIMW–MxCaRL	0.120	-0.074 to 0.315	0.225	0.475

Arch–tooth association	Std. β	Nominal 95% CI	p	FDR q
MxIMW–MxCaCL	0.243	0.058 to 0.428	0.010	0.070
MxIMW–MxCaMDW	0.133	-0.048 to 0.314	0.150	0.402
MxIMW–MxPM2RL	0.078	-0.114 to 0.269	0.427	0.662
MxIMW–MxPM2CL	-0.016	-0.200 to 0.167	0.860	0.937
MxIMW–MxPM2MDW	0.278	0.092 to 0.465	0.003	**0.042**
MxIMW–MxMMRL	0.017	-0.196 to 0.231	0.873	0.937
MxIMW–MxMDRL	-0.012	-0.247 to 0.222	0.920	0.937
MxIMW–MxMPRL	-0.109	-0.302 to 0.084	0.267	0.498
MxIMW–MxMCL	0.222	0.044 to 0.399	0.014	0.086
MxIMW–MxMMDW	0.285	0.116 to 0.453	0.001	**0.025**
MdAP–MdCIRL	0.296	0.115 to 0.476	0.001	**0.028**
MdAP–MdCICL	0.165	-0.034 to 0.365	0.104	0.340
MdAP–MdCIMDW	0.255	0.059 to 0.452	0.011	0.070
MdAP–MdCaRL	0.026	-0.189 to 0.242	0.810	0.911
MdAP–MdCaCL	0.290	0.098 to 0.481	0.003	**0.041**
MdAP–MdCaMDW	0.134	-0.059 to 0.328	0.174	0.426
MdAP–MdPM2RL	0.054	-0.120 to 0.228	0.541	0.735
MdAP–MdPM2CL	0.025	-0.168 to 0.218	0.798	0.911
MdAP–MdPM2MDW	0.169	0.008 to 0.331	0.040	0.169
MdAP–MdMMRL	0.105	-0.114 to 0.324	0.349	0.596
MdAP–MdMDRL	0.170	-0.044 to 0.385	0.120	0.371
MdAP–MdMCL	0.218	0.056 to 0.380	0.008	0.069
MdAP–MdMMDW	0.335	0.172 to 0.498	<0.001	**0.003**
MdICaW–MdCIRL	0.089	-0.120 to 0.299	0.404	0.651
MdICaW–MdCICL	0.139	-0.055 to 0.333	0.159	0.402
MdICaW–MdCIMDW	0.122	-0.077 to 0.322	0.230	0.475
MdICaW–MdCaRL	-0.069	-0.315 to 0.176	0.580	0.755
MdICaW–MdCaCL	0.100	-0.069 to 0.269	0.247	0.475
MdICaW–MdCaMDW	0.067	-0.184 to 0.318	0.600	0.763
MdICaW–MdPM2RL	-0.013	-0.187 to 0.161	0.886	0.937
MdICaW–MdPM2CL	-0.133	-0.345 to 0.079	0.218	0.475
MdICaW–MdPM2MDW	0.015	-0.166 to 0.196	0.870	0.937
MdICaW–MdMMRL	-0.053	-0.281 to 0.176	0.652	0.791
MdICaW–MdMDRL	0.014	-0.216 to 0.244	0.904	0.937
MdICaW–MdMCL	0.064	-0.144 to 0.273	0.544	0.735
MdICaW–MdMMDW	0.073	-0.105 to 0.251	0.424	0.662
MdPM2W–MdCIRL	0.238	0.055 to 0.421	0.011	0.070
MdPM2W–MdCICL	0.196	0.021 to 0.370	0.028	0.144
MdPM2W–MdCIMDW	0.070	-0.117 to 0.257	0.462	0.680
MdPM2W–MdCaRL	0.041	-0.164 to 0.247	0.693	0.832
MdPM2W–MdCaCL	0.132	-0.052 to 0.316	0.160	0.402
MdPM2W–MdCaMDW	-0.055	-0.270 to 0.159	0.613	0.770
MdPM2W–MdPM2RL	0.071	-0.092 to 0.233	0.395	0.646
MdPM2W–MdPM2CL	-0.144	-0.328 to 0.040	0.125	0.371
MdPM2W–MdPM2MDW	0.067	-0.101 to 0.235	0.435	0.662
MdPM2W–MdMMRL	0.074	-0.154 to 0.302	0.525	0.735
MdPM2W–MdMDRL	0.088	-0.136 to 0.312	0.442	0.663
MdPM2W–MdMCL	0.101	-0.082 to 0.284	0.279	0.511
MdPM2W–MdMMDW	0.080	-0.090 to 0.250	0.356	0.596

Arch–tooth association	Std. β	Nominal 95% CI	p	FDR q
MdIMW–MdCIRL	0.239	0.055 to 0.423	0.011	0.070
MdIMW–MdCICL	0.163	-0.007 to 0.334	0.061	0.219
MdIMW–MdCIMDW	-0.007	-0.188 to 0.174	0.937	0.946
MdIMW–MdCaRL	-0.060	-0.255 to 0.135	0.545	0.735
MdIMW–MdCaCL	0.117	-0.077 to 0.311	0.238	0.475
MdIMW–MdCaMDW	-0.074	-0.259 to 0.111	0.434	0.662
MdIMW–MdPM2RL	0.019	-0.138 to 0.176	0.809	0.911
MdIMW–MdPM2CL	-0.114	-0.304 to 0.077	0.242	0.475
MdIMW–MdPM2MDW	0.114	-0.053 to 0.282	0.182	0.426
MdIMW–MdMMRL	0.034	-0.194 to 0.262	0.770	0.894
MdIMW–MdMDRL	0.053	-0.175 to 0.280	0.650	0.791
MdIMW–MdMCL	0.091	-0.095 to 0.277	0.337	0.596
MdIMW–MdMMDW	0.078	-0.085 to 0.241	0.350	0.596

Note: Standardized coefficients (β) from patient-clustered Gaussian GEE models adjusted for skeletal class, age and sex; n = 111 patients (222 bilateral tooth observations). q, Benjamini–Hochberg FDR-adjusted p-value across all 108 associations. Nominal confidence intervals have not been adjusted for multiple testing. Bold type indicates FDR $q < 0.05$. Abbreviations are defined in Tables 3 and 5.

Supplementary Table S3. Intra-examiner reliability and method error for each of the 35 tooth and arch variables

Variable	ICC(2,1)	95% CI	Dahlberg error (mm)
**A. Maxillary tooth measurements**			
Crown length			
Central incisor	0.824	0.634–0.919	0.253
Canine	0.979	0.954–0.991	0.164
Second premolar	0.917	0.823–0.962	0.170
First molar	0.884	0.755–0.947	0.293
Root length			
Central incisor	0.964	0.922–0.984	0.264
Canine	0.984	0.958–0.994	0.325
Second premolar	0.967	0.919–0.986	0.272
First molar – mesiobuccal root	0.953	0.897–0.979	0.303
First molar – distobuccal root	0.981	0.952–0.992	0.205
First molar – palatal root	0.897	0.780–0.954	0.394
Mesiodistal width			
Central incisor	0.973	0.940–0.988	0.141
Canine	0.959	0.911–0.982	0.137
Second premolar	0.813	0.619–0.913	0.227
First molar	0.920	0.828–0.964	0.255
**B. Mandibular tooth measurements**			
Crown length			
Central incisor	0.939	0.867–0.973	0.193
Canine	0.935	0.860–0.971	0.237
Second premolar	0.900	0.786–0.955	0.180
First molar	0.870	0.728–0.940	0.216
Root length			
Central incisor	0.967	0.927–0.985	0.244
Canine	0.980	0.955–0.991	0.278
Second premolar	0.965	0.888–0.987	0.272
First molar – mesial root	0.960	0.899–0.983	0.268
First molar – distal root	0.912	0.807–0.961	0.411
Mesiodistal width			
Central incisor	0.895	0.777–0.952	0.163
Canine	0.883	0.754–0.947	0.178
Second premolar	0.933	0.856–0.970	0.172
First molar	0.894	0.775–0.952	0.296
**C. Maxillary dental arch measurements**			
Arch perimeter	0.944	0.877–0.975	0.176
Intercanine width	0.916	0.819–0.962	0.180
Inter-second premolar width	0.930	0.848–0.968	0.170
Intermolar width	0.925	0.838–0.966	0.213
**D. Mandibular dental arch measurements**			
Arch perimeter	0.891	0.771–0.950	0.201
Intercanine width	0.888	0.762–0.949	0.257
Inter-second premolar width	0.897	0.773–0.954	0.268
Intermolar width	0.910	0.808–0.959	0.241

Note: ICC, intraclass correlation coefficient; CI, confidence interval. ICC(2,1), two-way random-effects model, absolute agreement, single measurements; two repeated measurements of 25 scans. Dahlberg method errors are in millimeters.

Supplementary Table S4. Age- and sex-adjusted GEE sensitivity analyses of tooth morphology and dental arch dimensions across skeletal classes

Outcome	Wald χ^2 (df = 2)	p-value	FDR q-value
**Maxillary tooth morphology**			
MxCIRL	4.159	0.125	0.486
MxCICL	7.281	**0.026**	0.205
MxCIMDW	2.444	0.295	0.496
MxCaRL	5.537	0.063	0.314
MxCaCL	1.091	0.580	0.751
MxCaMDW	2.987	0.225	0.496
MxPM2RL	2.423	0.298	0.496
MxPM2CL	3.291	0.193	0.496
MxPM2MDW	2.677	0.262	0.496
MxMMRL	2.617	0.270	0.496
MxMDRL	0.502	0.778	0.879
MxMPRL	0.970	0.616	0.770
MxMCL	1.504	0.471	0.689
MxMMDW	1.340	0.512	0.689
**Mandibular tooth morphology**			
MdCIRL	0.155	0.925	0.925
MdCICL	10.522	**0.005**	0.091
MdCIMDW	1.410	0.494	0.689
MdCaRL	2.856	0.240	0.496
MdCaCL	12.191	**0.002**	0.079
MdCaMDW	0.217	0.897	0.924
MdPM2RL	6.694	**0.035**	0.205
MdPM2CL	5.129	0.077	0.337
MdPM2MDW	0.737	0.692	0.818
MdMMRL	0.430	0.807	0.882
MdMDRL	1.756	0.416	0.661
MdMCL	1.353	0.508	0.689
MdMMDW	2.445	0.294	0.496
**Dental arch dimensions**			
MxAP	2.993	0.224	0.496
MxICaW	3.409	0.182	0.496
MxPM2W	0.319	0.852	0.904
MxIMW	0.711	0.701	0.818
MdAP	6.932	**0.031**	0.205
MdICaW	9.024	**0.011**	0.128
MdPM2W	3.932	0.140	0.490
MdIMW	3.296	0.192	0.496

Note: Values represent omnibus skeletal-class effects from Gaussian generalized estimating equation (GEE) models including skeletal class (Class I, II, and III), age (continuous), and sex (categorical) as covariates. Patient-clustered robust (sandwich) standard errors and an independence working correlation structure were used. Wald χ^2 tests had 2 degrees of freedom. Each tooth outcome included 111 patients and 222 bilateral observations (74 observations per skeletal class); each arch outcome included 111 patient-level observations (37 per skeletal class). The Benjamini–Hochberg false discovery rate (FDR) procedure was applied jointly across all 35 adjusted omnibus p-values. Bold p-values indicate nominal $p < 0.05$; no outcome remained significant at FDR $q < 0.05$. Variable abbreviations are defined in Tables 3–5 of the main manuscript.
